# Supplementary material for: Dynalign II: common secondary structure prediction for RNA homologs with domain insertions
Source: Nucleic Acids Res. 2014 Nov 21;42(22):13939–48. doi: 10.1093/nar/gku1172 (PMC4267632; doi:10.1093/nar/gku1172)
Supplement: SUPPLEMENTARY DATA [file supp_gku1172_nar-02021-z-2014-File012.zip › manual/Text/refold.html]

RNAstructure Command Line Help: refold


|  |  |  |
| --- | --- | --- |
|  | RNAstructure Command Line Help refold | - Contents - Index |
| refold takes a previously folded sequence and predicts a new set of suboptimal structures from it. USAGE: refold <save file> <ct file> [options] Required parameters: <save file> \| The name of a binary folding save file containing folding information, generated by Fold.; <ct file> \| The name of a CT file to which output will be written. Options which don't require added values: -h, -H, --help \| Display the usage details message. Options which require added values: -m, -M, --maximum \| Specify a maximum number of structures. Note that suboptimal structures are generated until either the maximum number of structures are reached or the maximum percent difference is reached (below). Default is 20 structures.; -p, -P, --percent \| Specify a maximum percent difference in folding free energy change in suboptimal structures. Note that suboptimal structures are generated until either the maximum percent free energy difference is reached or until the maximum number of structures is reached (above). Default is 10 percent (specified as 10, not 0.1).; -w, -W, --window \| Specify a window size. Default is determined by the length of the sequence. References 1. Reuter, J.S. and Mathews, D.H. "RNAstructure: software for RNA secondary structure prediction and analysis." *BMC Bioinformatics>*, 11:129. (2010). 2. Mathews, D.H., Sabina, J., Zuker, M. and Turner, D.H. "Expanded sequence dependence of thermodynamic parameters provides improved prediction of RNA secondary structure." *J. Mol. Biol.*, 288:911-940. (1999). | | |
| Visit The Mathews Lab RNAstructure Page for updates and latest information. | | |
